# Combination of Endoscopic Resection and Radiofrequency Ablation for the Treatment of Esophageal Squamous Cell Neoplasia With Multiple Lugol-Voiding Lesions

**DOI:** 10.3389/fonc.2021.786015

**Published:** 2021-11-24

**Authors:** Zhihao Chen, Lizhou Dou, Yong Liu, Yueming Zhang, Shun He, Liyan Xue, Guiqi Wang

**Affiliations:** ^1^ Department of Endoscopy, National Cancer Center/National Clinical Research Center for Cancer/Cancer Hospital, Chinese Academy of Medical Sciences and Peking Union Medical College, Beijing, China; ^2^ Department of Pathology, National Cancer Center/National Clinical Research Center for Cancer/Cancer Hospital, Chinese Academy of Medical Sciences and Peking Union Medical College, Beijing, China

**Keywords:** endoscopic resection, radiofrequency ablation, multiple Lugol-voiding lesions, esophageal squamous cell neoplasia, background esophageal mucosa

## Abstract

**Background:**

Local recurrence of esophageal squamous cell neoplasia (ESCN) and metachronous ESCN was associated with severe background esophageal multiple Lugol-voiding lesions (LVLs) even though the primary early ESCNs were treated with endoscopic resection (ER). The aim of this study is to explore the feasibility and effectiveness of combination treatments of ER and radiofrequency ablation (RFA) in patients with early ESCNs with synchronous multiple LVLs.

**Methods:**

A total of 329 patients with early ESCNs and synchronous multiple LVLs received ER combined with RFA from September 2010 to September 2020. Clinical and pathological features and treatment outcomes were retrospectively reviewed using medical records. Factors associated with background esophageal multiple LVLs before combined treatment were analyzed.

**Results:**

The proportion of complete response (CR) was 96.7% after primary RFA, while 90.3% patients achieved CR for the last endoscopic examinations regardless if inside or outside the treatment area (TA). Degeneration of background esophageal multiple LVLs occurred in 70.2% of patients. The grade of background esophageal multiple LVLs before combined treatment was closely related to gender, smoking, and drinking. The incidence of metachronous ESCNs outside the TA of ER and local recurrence in the TA of ER was 3.9% and 1.2%, respectively.

**Conclusions:**

Prophylactic RFA treatment of multiple LVLs together with ER treatment of the primary ESCNs may be effective in reducing the incidence of metachronous ESCNs and local recurrence through improving the background esophageal mucosa.

## Introduction

Esophageal cancer is the sixth most common cause of cancer death worldwide (1). In the Asian region, esophageal squamous cell carcinoma (ESCC) is the major histologic type of the disease (2). Recent advances in image-enhanced endoscopy have enabled an early accurate diagnosis of esophageal squamous cell neoplasia (ESCN). Besides, chromoendoscopy with Lugol’s solution is highly sensitive for identifying dysplasia and superficial ESCC. The iodine-unstained mucosa reveals dysplastic changes of varying severity that often represent Lugol-voiding lesions (LVLs), whereas normal esophageal mucosa stains brown (3, 4). The size and the number of LVLs were associated with the risk for development of second primary cancers (5). Keisuke et al. (5) found that most of the LVLs with size less than 5 mm were diagnosed as non-neoplastic lesion (42.1%) or low-grade intraepithelial neoplasia (LGIN) (54.0%) according to pathological results of the biopsy or resected specimens. Patients with dysplasia and superficial ESCC (intramucosal or submucosal carcinoma) exhibit an overall 5-year survival rate of >90% (6).

The minimally invasive technique of endoscopic resection (ER), including endoscopic mucosal resection (EMR) and endoscopic submucosal dissection (ESD), is recommended to treat early ESCNs (7, 8). Previous studies have proved that ER for early ESCNs could achieve a high curative resection (CuR), and the long-term follow-up results showed that the 5-year cause-specific survival could reach 90% (7, 8), especially for patients with isolated lesions. However, ER alone in the treatment of early ESCNs with synchronous multiple LVLs often leads to excessive resection range due to unclear and irregular boundary, which has increased the surgical complications, such as bleeding, perforation, and stenosis. In addition, it was difficult to ensure negative lateral margin.

Recently, radiofrequency ablation (RFA) has been widely used in early ESCNs, especially for those flat lesions with irregular boundary, scattered or large range (9, 10). However, disadvantages also existed, such as small indication range, low complete response (CR) rate (ranges from 50% to 100%), and higher local recurrence or progressive rate (0%–50%) (11–13).

Both ER and RFA have their advantages and disadvantages in the treatment of early ESCNs. Guidelines regarding the management of early ESCNs with synchronous multiple LVLs have not been established. Therefore, we designed clinical study to explore the feasibility and effectiveness of combination treatments of ER and RFA for patients with early ESCNs with synchronous multiple LVLs.

## Materials and Methods

### Patients’ Characteristics and Indications

This retrospective study was conducted at the Cancer Institute and Hospital, Chinese Academy of Medical Sciences (CICAMS), Beijing, China, from September 2010 to September 2020. Patients were eligible if they met all of the following inclusion criteria: 1) aged 18–85 years; 2) high-resolution Lugol’s chromoendoscopy showing at least one unstained lesion (USL) containing high-grade intraepithelial neoplasia (HGIN) or early ESCC combined with multiple LVLs; 3) endoscopic ultrasound (EUS) with no submucosal invasion or lymphadenopathy; and 4) CT chest/abdomen (HGIN/ESCC patients) with no metastasis or lymphadenopathy (14, 15).

**Figure 1:**
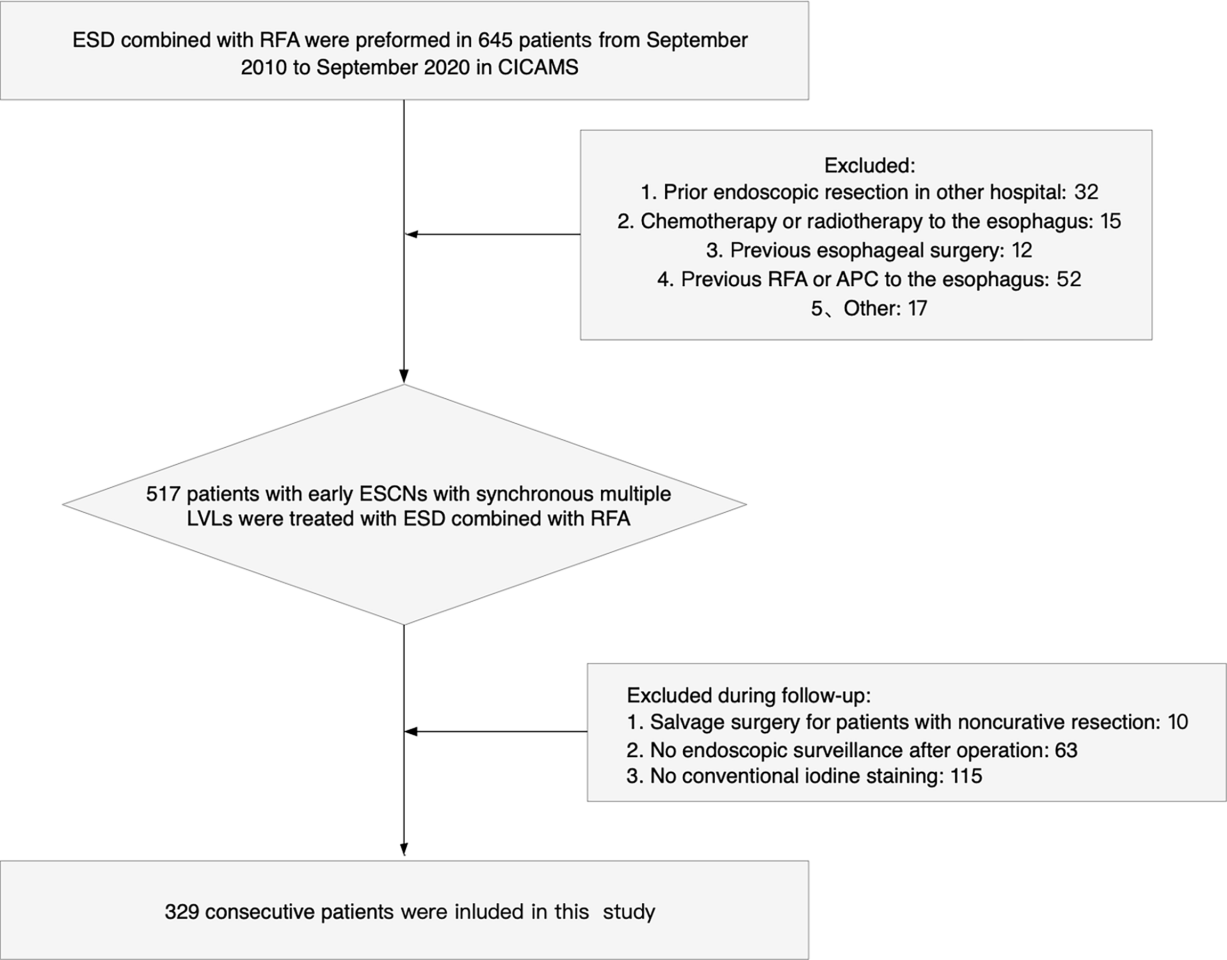
Flowchart depicting patient selection and follow-up in the study.

Patients were excluded if any one of the following exclusion criteria was present: 1) esophageal stricture preventing passage of therapeutic endoscope; 2) prior ER in other hospital; 3) previous RFA or argon plasma coagulation (APC) to the esophagus; 4) N- or M-positive ESCC; 5) chemotherapy or radiotherapy to the esophagus; 6) previous esophageal surgery, except fundoplication; 7) salvage surgery for patients with non-CuR; 8) no endoscopic follow-up; or 9) no Lugol’s solution staining during endoscopic follow-up (15–17). Flowchart depicting patient selection and follow-up in the study is presented in **Figure 1**.

### Lugol’s Chromoendoscopy

Endoscopy examinations were performed with Olympus GIF-H260, GIF-H260Z, GIF-H260J, GIF-H290, or GIF-HQ290 (Olympus, Tokyo, Japan) high-resolution endoscopes (Lucera Systems). After a conventional examination, approximately 10 ml of a 1.25% Lugol’s iodine solution was sprayed over the entire esophageal mucosa using a catheter, and the esophagus was inspected again. Mucosal biopsy specimens were collected from lesions that remain distinctly unstained by iodine (18).

**Figure 2:**
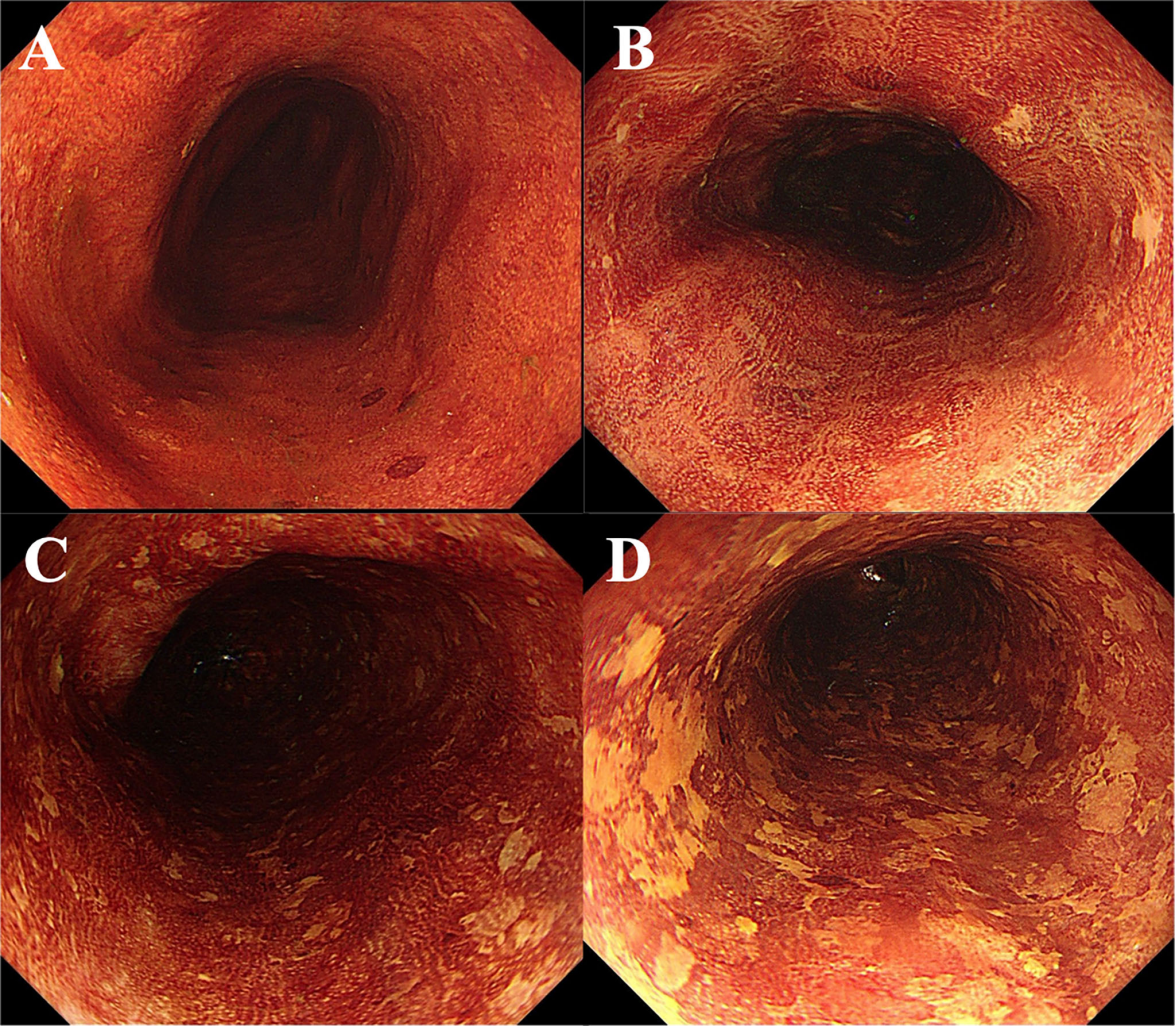
Endoscopic views of Lugol’s chromoendoscopy. **(A)** Absence of LVL. **(B)** Several (10 or less) small LVLs. **(C)** Many (more than 10) small LVLs. **(D)** Numerous irregular-shaped multiform LVLs. LVLs, Lugol-voiding lesions.

The pattern of LVLs was classified in accordance with the classification system proposed by Muto et al. (3) Patients were divided into four groups, based on the number and multiform pattern of LVLs in the background esophageal mucosa, namely, group A, no LVLs; group B, several (≤10) small LVLs; group C, many (>10) small LVLs; and group D, many irregular-shaped multiform LVLs (**Figure 2**). LVLs less than 5 mm in diameter were defined as small, whereas LVLs more than 5 mm in diameter and with irregular rims were defined as irregular-shaped multiform LVLs (**Figure 2**). Iodine staining was performed for every endoscopic examination, and experienced endoscopists assessed the pattern of LVLs according to the classification system above and recorded them in the endoscopic reports.

### Endoscopic Treatment and Histopathology Examination

Experienced endoscopists in CICAMS conducted all endoscopic procedures. All preoperative ESCN lesions with HGIN or worse histologic grade in biopsy specimens that met the Japan Esophageal Society guideline or the European Society of Gastrointestinal Endoscopy (ESGE) guidelines received ER (ESD or EMR) (14, 19). The ESD/EMR procedures included Lugol’s iodine staining, marking, submucosal injection, submucosal dissection/mucosal incision, and wound treatment (7, 8). The ER specimens were examined by at least two experienced pathologists based on the Japanese Classification of Esophageal Cancer (14, 20, 21).

However, the multiple LVLs containing LGIN outside the resected lesion were radiofrequency ablated through the Barrx™ Ablation System (Medtronic, Sunnyvale, CA, USA). The multiple LVLs of which the range was more than 3/4 of the circumference were treated with circumferential RFA regimens (BARXX^360^ system), while the scattered multiple LVLs were dealt with focal RFA (BARRX^90^, 3 × 12 J/cm^2^; Medtronic). The locations of all lesions and all treatment area (TA) (both ER and RFA) were determined by their distance from the incisor and their clock position on the circumferential esophagus. All data were recorded in the endoscopic reports.

### Follow-Up Strategy

After ER, the patients who had a CuR were subjected to upper-gastrointestinal (GI) endoscopy 3 and 12 months after ER, and annual surveillance was performed thereafter. For patients who had a non-CuR, surgical resection or chemoradiotherapy was carried out in the normal fashion, while some who refused additional treatment were followed-up with endoscopy, in addition to CT every 3 to 6 months. White light, narrow-band imaging (NBI) and Lugol’s iodine staining were required to find suspicious dysplastic lesions during the endoscopic follow-up, and biopsy sampling was done if there existed USLs or suspicious positive LVLs. Patients with lesions diagnosed as HGIN or worse histologic grade in biopsy specimens who met the Japan Esophageal Society guideline or the ESGE were treated with ER (14, 19). If subsequent biopsy samples showed LGIN, RFA treatment was repeated if patients agreed (22).

### Outcome Measures

The primary endpoint of this study was 1) the proportion of CR after primary RFA, defined as the absence of LGIN or worse in all TAs from biopsy samples at the 3-month visit; 2) degradation of background esophageal multiple LVLs, defined as the improvement of esophageal background mucosa at the last endoscopic follow-up after operation than before operation. Secondary study outcomes were 1) the proportion of patients without CR in the TA, defined as multiple LVLs persisting LGIN histologic grade in the TA at the last endoscopic follow-up; 2) the proportion of patients with recurrent disease in the TA of ER/progressive disease in the TA of RFA, defined as any USLs with HGIN or a worse histologic grade in the TA; 3) the proportion of patients with the development of metachronous LGIN or metachronous ESCN outside the TA; and 4) the total proportion of recurrent disease, progressive disease, metachronous disease, and LGIN persisting for the last endoscopic examination regardless if inside or outside the TA. Besides, the comparison of patient characteristics and clinical results in different groups of background esophageal multiple LVLs before combined treatment were explored in this study.

### Statistical Analyses

All statistical analyses were performed using SPSS 24.0 (SPSS, Chicago, IL, USA). Means ± SD or median deviation was computed for continuous variables, and frequencies and percentages were computed for categorical variables. The differences in the distribution between the groups were analyzed by using the *X* ^2^ test. The mean quantitative values were compared by using Student’s *t*-test, chi-square test, and Fisher’s exact test, as appropriate. The 5-year survival was assessed using the Kaplan–Meier survival curves. A *p*-value of <0.05 was considered statistically significant.

## Results

### Study Population and Lesion Outcomes

From September 2010 to September 2020, 329 patients with ESCNs and synchronous multiple LVLs who received ER combined with RFA were included in the baseline analysis. Baseline characteristics of the patients and operation details of endoscopic treatment are shown in **Table 1**.

**Table 1:** Patient characteristics and operation details of endoscopic treatment.

Age, mean ± SD	60.9 ± 7.4
Sex	
Male	285 (86.6%)
Female	44 (13.4%)
Alcohol drinking	212 (64.4%)
Drinking index (g*year)	6,307.0 ± 4,053.6
Cigarette smoking	212 (64.4%)
Smoking index	648.9 ± 499.2
History of any cancer in a first-degree relative	103 (31.3%)
History of any cancer	75 (22.8%)
History of head and neck neoplasm	38 (11.6%)
BMI, kg/m^2^, mean ± SD	23.5 ± 3.1
Resection methods	
ESD	300 (91.2%)
EMR/MBM	29 (8.8%)
Size of resected specimen (mm)	
Min–max	10–120
Mean ± SD	39.4 ± 14.1
Operation duration for ER (min)	
Median (min–max)	28 (6–150)
Operation duration for RFA (min)	
Median (min–max)	3 (1–10)
Procedure-related complication	
Bleeding	10 (3%)
Perforation	2 (0.6%)
Stricture	29 (8.8%)
Sessions of dilatation, median (range)	4 (1–20)

BMI, body mass index; ESD, endoscopic submucosal dissection; EMR, endoscopic mucosal resection; MBM, multiband mucosectomy; Min, minimum; Max, maximum; ER, endoscopic resection; RFA, radiofrequency ablation.

Besides, the tumor characteristics and pathological results of endoscopic treatment are also analyzed in this study (see **Table 2**). Three hundred patients were en bloc resected, and 80.9% of all included patients achieved CuR. Among 72 patients who did not achieve CuR, 12 patients (3.6%) underwent additional radiotherapy or chemotherapy or chemoradiotherapy (see **Supplementary Table**).

**Table 2:** Tumor characteristics and pathological results of endoscopic treatment.

Location within esophagus	
Upper	28 (8.5%)
Mid	187 (56.8%)
Lower	114 (34.7%)
Intraesophageal multiple cancers	82 (24.9%)
Circumferential extension of tumor	
Tumor ≤ 1/2	236 (71.7%)
1/2 < tumor ≤ 3/4	72 (21.9%)
3/4 < tumor ≤ 1	21 (6.4%)
Background esophageal multiple LVLs before combined treatment	
A	0 (0%)
B	82 (24.9%)
C	116 (35.3%)
D	131 (39.8%)
Tumor size (mm)	
Min–max	3-80
Mean ± SD	21.1 ± 12.7
Macroscopic type	
0–IIa	62 (18.8%)
0–IIb	229 (69.6%)
0–IIc	19 (5.8%)
0–IIa+IIc	19 (5.8%)
Depth of invasion	
EP	160 (48.6%)
LMP	71 (21.6%)
MM	50 (15.2%)
SM_1_ (≤200 μm)	16 (4.9%)
SM_2_ (>200 μm)	32 (9.7%)
Results of resection	
EnR	300 (91.2%)
R0	295 (89.7%)
CuR	266 (80.9%)

LVLs, Lugol-voiding lesions; ER, endoscopic resection; Min, minimum; Max, maximum; EP, epithelial; LMP, lamina propria; MM, muscularis mucosa; SM, submucosal; EnR, en-bloc resection; R0, completed resection; CuR, curative resection.

### Primary Outcomes

During the follow-up after primary RFA, the proportion of CR was 96.7%. Among 11 patients without CR in the TA of RFA, three patients (0.9%) with neoplastic progression received additional ER, two patients (0.6%) with persistent LGIN were treated with focal RFA and subsequently achieved CR, and the remaining six patients (1.8%) with persistent LGIN refused any treatment. Besides, the incidence of metachronous ESCNs was 3.9% outside the TA of ER, and the probability of local recurrence in the TA of ER was 1.2% (see **Table 3**).

**Table 3:** Clinical results of postoperative follow-up.

Follow-up, months, median (range)	37 (6–125)
CR rate after primary RFA	318 (96.7%)
Additional ER failed endoscopic treatment	17 (5.2%)
Neoplastic progression in the TA of RFA	3 (0.9%)
Local recurrence in the TA of ER	4 (1.2%)
Metachronous ESCN	10 (3%)
Additional RFA after primary RFA	42 (12.8%)
LGIN persisting in the TA of RFA	2 (0.6%)
LGIN persisting in the TA of ER	13 (4.0%)
Metachronous LGIN	27 (8.2%)
CR rate for the last EE	297 (90.3%)
LGIN persisting for the last EE	32 (9.7%)
LGIN persisting in the TA of RFA	6 (1.8%)
LGIN persisting in the TA of ER	15 (4.6%)
Metachronous LGIN	11 (3.3%)
Background esophageal multiple LVLs after combined treatment	
A	53 (16.1%)
B	141 (42.9%)
C	109 (33.1%)
D	26 (7.9%)
Degradation of background esophageal multiple LVLs	
0	98 (29.8%)
1	193 (58.7%)
2	37 (11.2%)
3	1 (0.3%)

CR, complete response; RFA, radiofrequency ablation; ER, endoscopic resection; TA, treatment area; ESCN, esophageal early squamous cell neoplasia; LGIN, low-grade intraepithelial neoplasia; EE, endoscopic examination; LVLs, Lugol-voiding lesions.

After combined treatment of ER and RA, degeneration of background esophageal multiple LVLs occurred in 70.2% of patients. The background esophageal multiple LVLs reduced by one grade in 193 patients (58.7%), 37 (11.2%) by two grades, and one (0.3%) by three grades. The comparison of background esophageal multiple LVLs before and after combined treatment is shown in **Table 4**.

**Table 4:** Comparison of background esophageal multiple LVLs before and after combined treatment.

		Background esophageal multiple LVLs after combined treatments				
		A	B	C	D	Total
Background esophageal multiple LVLs before combined treatment	B	38	44	0	0	82
	C	14	74	28	0	116
	D	1	23	81	26	131
	Total	53	141	109	26	329

LVLs, Lugol-voiding lesions; ER, endoscopic resection.

### Secondary Study Outcomes

In this study, 90.3% patients achieved CR for the last endoscopic examinations regardless if inside or outside the TA. However, inside the TA of ER, the proportion of local recurrence was 1.2%, while 8.6% of all included patients were found local LGIN recurrence, and 13 of them agreed to receive focal RFA. The number of patients with metachronous ESCNs and LGIN outside the TA was 10 (3%) and 27 (8.2%) respectively (see **Table 3**).

The total proportion of progressive diseases (HGIN or a worse histologic grade) regardless if inside or outside the TA was 5.1%, and all of them were treated with additional ER. There were 71 patients with recurrence or residual LGIN after the combination treatments of ER and primary RFA during follow-up, 42 of whom received additional RFA, and most of them (39/42, 92.9%) were completely healed, while the remaining selected regular endoscopic surveillance.

### Comparison of Different Groups of Background Esophageal Multiple Lugol-Voiding Lesions Before Combined Treatment

According to the grading standard of background esophageal multiple LVLs before combined treatment, 82 (24.9%) patients were assessed as Grade B, 116 (35.3%) patients as Grade C, and 131 (39.8%) as Grade D. Results of the comparison of patient characteristics and clinical results among different groups are shown in **Table 5**. There were no significant differences in age, body mass index (BMI), history of any cancer, history of any cancer in a first-degree relative, and additional RFA after primary RFA.

**Table 5:** Comparison of patient characteristics and clinical results among different groups of background esophageal multiple LVLs.

	Background esophageal multiple LVLs before combined treatment			
	B (82)	C (116)	D (131)	*p*
Age, mean ± SD	62.24 ± 8.08	60.80 ± 7.79	60.15 ± 6.92	0.157
Sex (male)	53 (64.6%)	103 (88.8%)	129 (98.5%)	<0.001
BMI, kg/m^2^, mean ± SD	23.49 ± 3.11	23.65 ± 3.09	23.41 ± 3.01	0.839
Intraesophageal multiple cancers	11 (13.4%)	23 (19.8%)	48 (36.6%)	<0.001
History of any cancer	13 (15.9%)	27 (23.3%)	35 (26.7%)	0.182
History of head and neck neoplasm	2 (2.4%)	12 (10.3%)	24 (18.3%)	0.002
History of any cancer in a first-degree relative	29 (35.4%)	34 (29.3%)	40 (30.5%)	0.644
Cigarette smoking	32 (39.0%)	74 (63.8%)	106 (80.9%)	<0.001
Alcohol drinking	29 (35.4%)	72 (62.1%)	111 (84.7%)	<0.001
CuR rate	84.1%	82.8%	70.2%	0.019
CR rate after primary RFA	100%	97.4%	93.9%	0.046
Additional ER failed endoscopic treatment	3 (3.7%)	2 (1.7%)	12 (9.2%)	0.024
Additional RFA after primary RFA	5 (6.1%)	17 (14.7%)	21 (16.0%)	0.092
CR rate for the last EE	98.8%	92.2%	83.2%	0.001
Degradation of background esophageal multiple LVLs				<0.001
0	44 (53.7%)	28 (24.1%)	26 (19.8%)	
1	38 (46.3%)	74 (63.8%)	81 (61.8%)	
2	0	14 (12.1%)	23 (17.6%)	
3	0	0	1 (0.8%)	

LVLs, Lugol-voiding lesions; ER, endoscopic resection; BMI, body mass index; CuR, curative resection; RFA, radiofrequency ablation; CR, complete response; EE, endoscopic examination.

The grade of background esophageal multiple LVLs before combined treatment was closely related to gender, smoking, and drinking. In Grade D, 98.5% patients were male, 80.9% were smokers, and 84.7% were drinkers, which were significantly higher compared with 88.8%, 63.8%, and 62.1% in Grade C and 64.6%, 39.0%, and 35.4% in Grade B, respectively (*p* < 0.001). Compared with the patients Grade B and Grade C, the patients in Grade D were more likely to have multiple synchronous ESCNs (13.4% *vs*. 19.8% *vs*. 36.6%, *p* < 0.001). Besides, the proportion of patients with synchronous or metachronous head and neck neoplasm in Grade C and Grade D was significantly higher than that in Grade B (10.3% *vs*. 18.3% *vs*. 2.4%, *p* = 0.002).

In terms of treatment, Grade B and Grade C can achieve both higher CuR rate and higher CR rate after primary RFA than Grade D (84.1% *vs*. 82.8% *vs*. 70.2%, *p* = 0.019; 100% *vs*. 97.4% *vs*. 93.9%, *p* = 0.046). However, Grade C and Grade D were associated with greater benefits in combination therapy of ER and RFA, as evidenced by a significantly higher percentage of background esophageal multiple LVL degradation than Grade B (75.9% *vs*. 80.2% *vs*. 46.3%, *p* < 0.001).

### Follow-Up Outcomes

Adverse events occurred in 38 patients (11.6%), namely, bleeding (n = 10, 3%), suspicious microperforation (n = 2, 0.6%), and stricture (n = 29, 8.8%). Both delayed bleeding and postoperative esophageal stenosis occurred in two patients, and another one patient suffered from both delayed bleeding and perforation. All adverse events were handled endoscopically, without leaving any serious consequences.

During a median follow-up period of 37 months (range 6–125 months), nine patients (2.7%) died, and only one of them died of ESCC. Thus, the 5-year overall survival and 5-year cause-specific survival were 97.3% and 99.7%, respectively.

## Discussion

Previous studies (3, 5, 18) have shown that smoking and drinking are closely related to the background esophageal multiple LVLs. This study confirmed the conclusion above and also found that the heavier the smoking and drinking is, the more severe the background esophageal mucosa will be. However, the management of early ESCNs with dense, scattered, and irregular background esophageal multiple LVLs has been bothering clinicians for a long time. Guidelines regarding this area have not been established. This research is the first to explore the feasibility and effectiveness of the combined treatment of ER and RFA in patients with early ESCNs and synchronous multiple LVLs.

Several studies (3, 5, 18) have proved that the severity of background esophageal multiple LVLs is closely correlated with synchronous or metachronous ESCNs, and head and neck tumor. This study also confirmed that the patients in Grade D were more likely to have a second primary squamous cell carcinoma (synchronous or metachronous) in the esophagus, head, and neck than the patients in Grade C and Grade B (36.6% *vs*. 19.8% *vs*. 13.4%, *p* < 0.001, 18.3% *vs*. 10.3% *vs*. 2.4%, *p* = 0.002). Even after ER treatment of the primary early ESCNs, Katada et al. (23) and Urabe et al. (24) respectively demonstrated that local recurrence and metachronous ESCNs were associated with severe background esophageal mucosa. A cohort study targeted at patients who had undergone ER for early ESCC demonstrated a strong association between the cumulative incidence of multiple metachronous ESCNs and the grade of esophageal LVLs (25). Urabe et al. (26) then established a predictive model and have proved that multiple LVLs were one of the independent risk factors for multiple metachronous development in early ESCN patients after ER treatment. Besides, Suzuki et al. (27) demonstrated in a controlled trial that additional chemoradiotherapy for ESCC patients with non-CuR can reduce the incidence of metachronous ESCC by improving the background esophageal mucosa. Therefore, it seems necessary to conduct prophylactic treatment of multiple LVLs while delivering ER treatment of the primary ESCNs.

In clinical practice, for patients with poor background esophageal mucosa who received ER treatments, the incidence of metachronous ESCNs was significantly higher than that of patients with isolated ESCNs (24, 26). The rate of developing metachronous ESCC and local recurrence in the ESCNs patients after ER was reported to range from 12% to 35% and from 3.9% to 20%, respectively (22, 23, 26, 28–31). Although RFA has been proved to have a relatively large therapeutic advantage in background esophageal multiple LVLs with dispersion, and irregular and unclear boundary, the indications for RFA in the treatment of ESCNs are very limited (9, 10, 12, 16). Yu et al. (12) have demonstrated that RFA was not suitable for USL with a pink-color sign or ESCC in the biopsy samples. In addition, CR could only reach 86% in 5 years, and 14% of lesions showed local recurrence or pathological progression during the follow-up (12). However, in this study, the incidence of metachronous ESCNs (3.9%) outside the TA of ER and the probability of local recurrence in the TA of ER (1.2%) were lower than those in the present studies (22, 23, 26, 28–31). The decrease in the incidence of metachronous ESCNs and local recurrence may be attributed to the LGIN treated with the primary RFA and emerging LVLs treated with additional RFA during subsequent follow-up. However, another 9.7% of the included patients were still found LGIN persisting in the last endoscopic examination, and the patients refused further intervention. These lesions may be a potential risk factor for metachronous ESCNs (5.1%) outside the TA of ER and local recurrence in the TA of ER (4.6%). Thus, more close endoscopic follow-up is required for timely detection and management of early lesions for these patients. Moreover, through the combined therapy of ER and RFA, the background esophageal mucosa was significantly improved postoperatively (70.2%), especially for patients with preoperative background esophageal mucosa assessed as Grade D (80.2%). Besides, compared with that of previous studies (6, 7, 15, 32), the additional RFA for multiple LVLs on the basis of ER of early ESCNs did not increase the incidence of postoperative complications. Thus, this combination treatments for patients with early ESCNs and synchronous multiple LVLs not only can give full play to the advantages of complete resection of the primary lesions and pathological evaluation postoperatively but also can leverage the advantages of RFA in the treatment of dense, scattered, and irregular multiple LVLs.

During the follow-up after the combination treatments (median 37 months, range 6–125 months), there were 71 patients with recurrence or residual LGIN, 42 of whom received additional RFA, and most of them (39/42, 92.9%) were completely healed, while the remaining selected regular endoscopic surveillance, and no lesion progression was observed. In addition, the probability of persisting LGIN and metachronous ESCN in TA of primary RFA was lower than that outside the TA of ER and primary RFA (2.4% *vs*. 11.5%, *p* < 0.001, 0.9% *vs*. 3%, *p* = 0.05) (see **Table 3**), which indicated that RFA treatment for multiple LVLs may prevent the occurrence of metachronous ESCNs. What is more, the 5-year overall survival rate and 5-year cause-specific survival rate in this study were 97.3% and 99.7%, respectively, which were slightly higher than those of patients treated with ER alone in the previous studies (6, 33).

There are also several limitations in this study. Firstly, referral bias may not be excluded in this retrospective analysis, which was based on records at a single center and without a control group, due to the different and unmatched inclusion criteria between the experimental group and control group. Secondly, the median follow-up time of the patients was 37 months, which may be not long enough for evaluation of the long-term outcomes. The most important limitation of this study is that endoscopists were unlikely to biopsy all LVLs in the esophagus, especially for scattered and small LVLs, but experienced endoscopists tried their best to examine the esophagus accurately by using white light, NBI, and iodine staining; and all suspected dysplastic lesions were biopsy sampled. Last but not least, this study is the first research to explore the clinical effectiveness of prophylactic treatment of multiple LVLs together with ER treatment of the primary ESCNs with large sample size.

In summary, this study preliminarily revealed that the combined therapy of RFA and ER in the treatment of patients with early ESCNs and synchronous multiple LVLs can reduce the incidence of metachronous ESCNs and local recurrence through improving the background esophageal mucosa and may slightly improve the 5-year cause-specific survival rate. However, additional prospective, randomized, controlled, multicenter studies with a larger number of cases will be needed to confirm the feasibility and effectiveness of combination therapy in the treatment of patients with early ESCNs and synchronous multiple LVLs.

## Data Availability Statement

The original contributions presented in the study are included in the article/**Supplementary Material**. Further inquiries can be directed to the corresponding author.

## Ethics Statement

The studies involving human participants were reviewed and approved by The Ethics Committee of National Cancer Center/Cancer Hospital, Chinese Academy of Medical Science and Peking Union Medical College (Approval Number: 09-76/371). The patients/participants provided their written informed consent to participate in this study.

## Author Contributions

ZC drafted the protocol, extracted the data, performed the statistical analyses, performed the literature search, and drafted the article. LD, YL, YZ, SH, and LX drafted the protocol, performed the literature search, and revised the article. GW directed the writing, review, and revision of the article. ZC is the first author. All authors contributed to the article and approved the submitted version.

## Funding

This work was supported by grants from the National Key Research and Development Program of China (grant number 2016YFC1302801), National Key Research and Development Program of China (grant number 2017YFC0908300), Beijing Science and Technology Planning Project (CN) (grant number D17110002617002), CAMS Innovation Fund for Medical Sciences (CIFMS) (grant number 2016-I2M-1-001), CAMS Innovation Fund for Medical Sciences (CIFMS) (grant number 2017-I2M-1-106), CAMS Innovation Fund for Medical Sciences (CIFMS) (grant number 2019-I2M-2-004), Sanming Project of Medicine in Shenzhen (grant number SZSM201911008), and PUMC Youth Fund and the Fundamental Research Funds for the Central Universities (grant number 2017320012).

## Conflict of Interest

The authors declare that the research was conducted in the absence of any commercial or financial relationships that could be construed as a potential conflict of interest.

## Publisher’s Note

All claims expressed in this article are solely those of the authors and do not necessarily represent those of their affiliated organizations, or those of the publisher, the editors and the reviewers. Any product that may be evaluated in this article, or claim that may be made by its manufacturer, is not guaranteed or endorsed by the publisher.

## Supplementary Material

Supplementary Table Postoperative pathological characteristics of patients with noncurative resection who received additional treatment.
