# Supplementary material for: Combination of Endoscopic Resection and Radiofrequency Ablation for the Treatment of Esophageal Squamous Cell Neoplasia With Multiple Lugol-Voiding Lesions
Source: Front Oncol. 2021 Nov 24;11:786015. doi: 10.3389/fonc.2021.786015 (PMC8651547; doi:10.3389/fonc.2021.786015)
Supplement: Supplementary Table — Postoperative pathological characteristics of patients with noncurative resection who received additional treatment. [file DataSheet_1.pdf]

Supplementary table Postoperative pathological characteristics of patients with noncurative resection who received additional treatment

	Pathological results							Results of resection			
	Horizontal margin	Vertical margin	Vessel invasion	Lymphatic invasion	Nerve invasion	Depth of invasion	Histologic type	EnR	R0	CuR	Treatment
1	-	-	-	-	-	SM_2	Undifferentiated	Yes	Yes	No	Radiotherapy
2	-	-	-	-	-	SM_2	Differentiated	Yes	Yes	No	Radiotherapy
3	-	-	-	-	-	SM_2	Undifferentiated	Yes	Yes	No	Chemoradiotherapy
4	-	-	-	-	-	SM_2	Undifferentiated	Yes	Yes	No	Radiotherapy
5	-	-	-	-	-	SM_2	Undifferentiated	Yes	Yes	No	Chemoradiotherapy
6	-	-	-	-	-	SM_2	Differentiated	Yes	Yes	No	Radiotherapy
7	-	-	-	+	-	SM_2	Undifferentiated	Yes	Yes	No	Radiotherapy
8	-	-	-	-	-	SM_1	Differentiated	No	No	No	Radiotherapy
9	-	-	-	+	-	SM_2	Undifferentiated	Yes	Yes	No	Radiotherapy
10	-	-	-	+	-	SM_1	Undifferentiated	Yes	Yes	No	Radiotherapy
11	-	-	-	+	-	MM	Differentiated	Yes	Yes	No	Radiotherapy
12	-	-	+	+	-	SM_2	Undifferentiated	Yes	Yes	No	Chemotherapy

MM muscularis mucosa; *SM_1* submucosa (≤200μm); *SM_2* submucosa (> 200μm); *EnR* En-bloc resection; *R0* Completed resection; *CuR* curative resection;
